# PTSD, depression and anxiety in Ebola virus disease survivors in Beni town, Democratic Republic of the Congo

**DOI:** 10.1186/s12888-021-03343-7

**Published:** 2021-07-08

**Authors:** Célestin Kaputu-Kalala-Malu, Eric Mafuta Musalu, Tim Walker, Olga Ntumba-Tshitenge, Steve Ahuka-Mundeke

**Affiliations:** 1 Department of Neurology, Centre Neuropsychopathologique (CNPP), Kinshasa University Teaching Hospital, Kinshasa, Democratic Republic of Congo; 2 School of Public Health, Kinshasa University teaching Hospital, Kinshasa, Democratic Republic of Congo; 3 School of Medicine and Public Health, University of Newcastle, Callaghan, New South Wales Australia; 4 Institut National de Récherche Biomédicale, Kinshasa, Democratic Republic of Congo & Microbiology service, Kinshasa University Teaching Hospital, Kinshasa, Democratic Republic of Congo

**Keywords:** PTSD, Depression, Anxiety, Ebola virus disease survivors, DRC

## Abstract

**Background:**

Ebola Virus Disease (EVD) is a deadly and feared infectious disease, which can be responsible of debilitating physical and psychological sequelae in survivors including depression and anxiety disorders. Unfortunately, there are scarce data on survivor sequelae in Democratic Republic of the Congo. So this study assessed PTSD, depression and anxiety symptoms among EVD survivors enrolled in the follow-up program of the psychosocial care team of Beni town’s general hospital.

**Methods:**

A cross-sectional study used consecutive sampling to recruit 144 Ebola virus disease survivors who came for follow up from October 23 to November 13; 2019. Basic socio-demographic data, presence of headache and short-term memory function were assessed. The Post-traumatic Checklist Scale and Hospital Anxiety and Depression Scale were used to assess psychological burden among participants. Descriptive statistics were used to summarized data and Pearson’s or likelihood chi-square were used to test association between psychiatric disorders and associated factors.

**Results:**

The prevalence of PTSD, depression and anxiety was 24.3, 24.3 and 33.3% respectively. Being male (OR = 0.42, 95% CI: 0.16, 0.95, *p* = 0.049), suffering from persistent headache (OR = 2.62, 95% CI: 1.12, 6.14, *p* = 0.014), losing a loved one because of EVD (OR: 2.60, 95% CI: 1.11, 6.15, *p* = 0. 015) and being young − 18-24 years - (OR: 0. 261, 95% CI: 0. 08, 0.82, *p* = 0,026) were statistically associated with PTSD diagnosis. Having short-term memory impairment and suffering from persistent headache were statistically associated with depression and anxiety diagnoses (OR = 2.44, 95% CI: 1.03, 5.82, *p* = 0.026); (OR = 2.24, 95% CI: 1.04, 4.85, *p* = 0.025); (OR = 2.62, 95% CI: 1.12, 6.14, *p* = 0.014); (OR = 2.31, 95% CI: 1.06, 5.01, *p* = 0.020).

**Conclusion:**

The prevalence of PTSD, depression and anxiety is high among EVD survivors. Development of specialized psychiatric services to sustain psychiatric and psychological health amongst survivors in the cultural context of the Eastern part of the DRC should be considered by the teams fighting against EVD in the DRC.

## Background

The 10th Ebola Virus disease (EVD) outbreak, declared in the North- Kivu and Ituri provinces, Democratic Republic of the Congo, on 1 August 2018, was the second largest in the world after the one that affected Western Africa between 2013 and 2016 [1, 2]. By the time it was declared over, on 25 June, 2020, it had affected 3470 and killed 2287 people, with 1171 survivors (https://www.who.int/news-room/detail/25-06-2020-10th-ebola-outbreak-in-the-democratic-republic-of-the-congo-declared-over-vigilance-against-flare-ups-and-support-for-survivors-must-continue). It was particularly challenging to manage this outbreak as it took place in an area where long-lasting conflict among countless armed groups is ongoing (https://www.who.int/news-room/detail/25-06-2020-10th-ebola-outbreak-in-the-democratic-republic-of-the-congo-declared-over-vigilance-against-flare-ups-and-support-for-survivors-must-continue) [3, 4]**.** This unstable situation affects the mental health of those living in these provinces, [5] which also lack proper mental health services [6, 7]. Previous studies suggest that EVD survivors are likely to present with neuropsychological disorders and posttraumatic stress reactions [8–10].

This study therefore aimed to determine the prevalence of depression, anxiety and post-traumatic stress disorder among EVD survivors. In addition, it sought to assess the relationship between these psychiatric disorders and other comorbidities among EVD survivors.

## Methods

### Sampling

This cross-sectional study was conducted from October 26 to November 13, 2019 in the psychosocial care service located in Beni town’s General Hospital. The psychosocial care team for EVD survivors of Beni town, North-Kivu province, Democratic Republic of the Congo (DRC) examined and evaluated 179 representing almost 15.3% of the survivors who had original discharge certificates from the Ebola treatment unit (ETU). It was a convenience sample of EVD survivors who presented for follow-up. This psychosocial care team was staffed by 8 mental health professionals. EVD survivors were assessed according to a pre-established consultation schedule which consisted of a monthly appointment, recurring over a period of 12 months. The duration of the assessment was approximately 6 h per patient. Each EVD survivor was first reviewed by a nurse for registration and vital signs measurements. In a second review, the patient was examined by a general practitioner for follow-up of any organic symptoms, either linked or not to EVD before being examined by mental health professional.

### Main outcomes measures and data collection

During the neuropsychiatric consultation, data were recorded using a predetermined questionnaire (one questionnaire per patient) consisting of basic socio-demographic information, the chief complaint, marital status, education level, discharge date from the ETU, presence of headache attributed to EVD by the patient, presence of short-term memory (STMI) attributed to EVD by the patient, and the presence of PTSD, depression and anxiety symptoms.

The French version of the Posttraumatic Checklist Scale (PCLS) [11, 12] was used as an assessment tool to screen for posttraumatic stress disorder. A score of 44 was used as a cut-off for this screening tool for the diagnosis of PTSD. The Hospital Anxiety and Depression Scale (HADS) was used for the diagnosis of anxiety and depression disorders. This scale has already been validated in general practice patients [13] and in non-hospitalized psychiatric patients [14]. The threshold for the diagnosis of anxiety and depression disorders was 8 [14]. The EVD survivors was considered as having STMI when he recognized to having trouble to remember things he heard, saw or did recently. Specifically, it was asked to confirm if they used to forget where theirs mobile phone was when they wanted to use it.

Due to the high illiteracy rate among EVD survivors, the neuropsychiatrist was responsible for helping patients understand some questionnaire’s items. This is one of the reasons why patients eligible for inclusion were ≥ 18 years of age, (average age of graduation from secondary school in the DRC). The administration of these two assessments lasted 20 to 30 min without help and 30 to 40 min with the help of the senior neuropsychiatrist. As the same words can be used for multiple concepts in Swahili, another commonly used language in the area the study was conducted, it was therefore important that most important concepts being translated and used by mental health professionals when any help was needed. The terms: « frightened feeling », « worrying thoughts », « feeling cheerfull » and « feelings of panic » were respectively translated by «hoga », « mafikiri », « ku kuwa na hali nzuri » and « wasi wasi » as far as HADS was concerned. The term « stressfull experience » was translated by « kipindi cha mafadhaiko / mafazaiko » when PCLS was used.

### Statistical analysis

Data were recorded on forms designed using Epi-Data 7.1.5 software and analyzed using Statistical Package for Social Science software version 21.0 (IBM Inc., Chicago, IL, USA). Data from categorical variables were summarized using proportions and percentages, and from numerical variables using mean with standard deviation if normally distributed and using median with interquartile range if not normally distributed. The Pearson χ^**2**^ test was used to compare proportions and determine the association between categorical variables. Means were compared using the student t test as two independent sample groups were (those who present psychiatric disorder and those who have not presented them) compared. The significance threshold was set at *p* = 0. 05.

## Results

Over the period of this study, 179 EVD survivors were evaluated by the psychosocial care team. A total of 144 (80.4%) aged 18 years or more were enrolled in this study.

### Baseline characteristics of EVD survivors

The majority of EVD survivors were female (63. 2%); with 64% of survivors under the age of 40. Most survivors had a low level of education (40.3% had never been to school, 37.5% had only primary level education). Forty-five percent of EVD survivors had lost at least one family member (parent, child, brother or sister) due to EVD (Table 1).

**Table 1:** Baseline characteristics

	EVD survivors	Percentage (*N* = 144)
**SEX**		
Female	91	63. 2
**AGE GROUPS (years)**		
18–24	30	20. 8
25–39	62	43. 1
≥ 40	52	36. 1
**EDUCATION**		
None	58	40. 3
Primary	54	37. 5
Secondary	22	15. 3
Post-secondary	10	6. 9
**MARITAL STATUS**		
Maried	90	62. 5
single	32	22. 2
Widower/ widow	20	13. 9
Divorced	2	1.4
**FAMILY MEMBER LOSS, yes**	65	45. 1
**HEADACHE, Yes**	53	36. 8
**STMI, Yes**	71	49. 3

*EVD* Ebola Virus disease, *PTSD* Posttraumatic stress disorders, *STMI* Short-term memory impairment

### Clinical findings

**Fig. 1:**
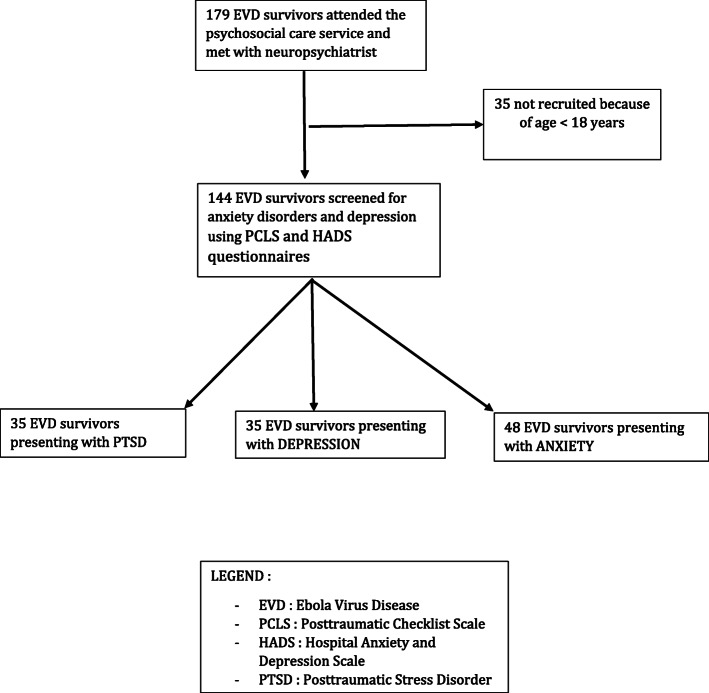
Flowchart showing study sampling and psychiatric disorders diagnosis

We noted that symptoms consistent with PTSD, depression and anxiety were found among survivors of EVDs in 24.3, 24.3 and 33.3% respectively (Fig. 1; Flowchart). Furthermore, 36.8% of patients had persistent headaches attributed to EVD, and 49.3% had reported persistent short-term memory they attributed to EVD.

Males survivors were less likely to experience post-traumatic stress disorder (OR = 0.42, 95% CI: 0.16, 0.95, *p* = 0.049); and anxiety (OR = 0.45, 95% CI: 0.19, 0.96, *p* = 0.038) than females. Survivors who lost a family member (parent, brother, sister and/or child) were twice as likely to have PTSD than those who did not lose a loved one (OR: 2.60, 95% CI: 1.11, 6.15, *p* = 0.015).

There was a statistically significant relationship between PTSD and persistent headache. EVD survivors suffering from persistent headache were twice as likely to have PTSD compared to headache-free survivors (OR = 2.62, 95% CI: 1.12, 6.14, *p* = 0.014).

This study revealed a statistically significant relationship between age and PTSD (OR: 0.261, 95% CI: 0.08, 0.82, *p* = 0.026). The younger the EVD survivor was, the greater was the risk for him or her to suffer from PTSD. Further analysis showed that it was 4 times less likely for a patient aged 40 or older to present with PTSD than for those aged 18–24 years (Table 2).

**Table 2:** Psychiatric diagnosis compared to survivor’s baseline characteristics

		PTSD			Depression			Anxiety		
		*N* = 144			*N* = 144			*N* = 144		
		Yes (*N* = 35)	No (*N* = 109)		Yes (*N* = 35)	No (*N* = 109)		Yes (*N* = 48)	Yes (*N* = 96)	
**SEX**	Male	8 (22.9%)	45 (41.3%)	*p* = 0. 049 OR = 0. 42 (CI: 0.16–0.95)	10 (28.6%)	43 (39.4%)	*p* = 0. 246	12 (25.0%)	41 (42.7%)	*p* = 0. 038 OR = 0.45 (CI: 0.19–0.96)
	Female	27 (77.1%)	64 (58.7%)		25 (71.4%)	66 (60.6%)		36 (75.0%)	55 (57.3%)	
**AGE GROUPS (years)**	18–24	10 (28.6%)	20 (18.3%)	1 OR = 0. 884 (CI: 0. 32–2.49) ^a^ OR = 0. 261 (CI: 0.08–0.82) ^b^ *p* = 0. 026	6 (17.1%)	24 (20.0%)	*p* = 0. 148	11 (22.9%)	19 (19.8%)	*p* = 0. 05
	25–39	19 (54.3%)	43 (39.4%)		20 (57.1%)	42 (38.5%)		26 (54.2%)	36 (37.5%)	
	**≥** 40	6 (17.1%)	46 (42.2%)		9 (25.7%)	43 (39.4%)		11 (22.9%)	41 (42.7%)	
**EDUCATION**	None	14 (40.0%)	44 (40.4%)	*p* = 0. 617^c^	14 (40.0%)	44 (40.4%)	*p* = 0.254^c^	20 (41.7%)	38 (39.6%)	*p* = 0. 090
	Primary	13 (37.1%)	41 (37.6%)		16 (45.7%)	38 (34.9%)		18 (37.5%)	35 (37.5%)	
	Secondary	7 (20.0%)	15 (13.8%)		5 (14.3%)	17 (15.6%)		10 (20.8%)	12 (12.5%)	
	Postsecondary	1 (2.9%)	9 (8.3%)		0 (0.0%)	10 (9.2%)		0 (0.0%)	10 (10.4%)	
**MARITAL STATUS**	maried	18 (51.4%)	72 (66.1%)	*p* = 0. 102^c^	22 (62.9%)	68 (62.4%)	*p* = 0.489^c^	27 (56.2%)	63 (65.6%)	*p* = 0. 403^c^
	Single	8 (22.9%)	22 (22.0%)		6 (17.1%)	26 (23.9%)		12 (25.0%)	20 (20.8%)	
	Widower / widow	9 (25.7%)	11 (10.1%)		7 (20.0%)	13 (11.9%)		18 (18.8%)	11 (11.5%)	
	divorced	0 (0.0%)	2 (1.8%)		0 (0.0%)	2 (1.8%)		0 (0%)	2 (2.1%)	
**STMI**	Yes	22 (62.9%)	49 (45.0%)	*p* = 0. 065	23 (65.7%)	48 (44.0%)	*p* = 0.026 OR = 2.44 (CI: 1.03–5.82)	30 (62.5%)	41 (42.7%)	*p* = 0.025 OR = 2.24 (CI: 1.04–4.85)
	No	13 (37.1%)	60 (55.0%)		12 (34.3%)	61 (56.0%)		18 (37.5%)	55 (57.3%)	
**HEADACHE**	Yes	19 (54.3%)	34 (31.2%)	*p* = 0. 014 OR = 2. 62 (CI: 1.12–6.14)	19 (54.3%)	34 (31.2%)	*p* = 0. 014 OR = 2.62 (CI: 1.12–6.14)	24 (50.0%)	29 (30.2%)	*p* = 0.020 OR = 2. 31 (CI: 1.06–5.01)
	No	16 (45.7%)	75 (68.8%)		16 (45.7%)	75 (68.8%)		24 (50.0%)	67 (69.8%)	
**F.M. L**	Yes	22 (62.9%)	43 (39.4%)	*p* = 0. 015 OR = 2. 60 (CI: 1.11–6.15)	19 (54.3%)	46 (42.2%)	*p* = 0. 211	23 (47.9%)	42 (43.8%)	*p* = 0. 636
	No	13 (37.1%)	66 (60.6%)		16 (45.7%)	63 (57.8%)		24 (52.1%)	54 (56.2%)	
	**TIME ELAPSED SINCE ETU DISCHARGE (mean, SD) / months**	6.1 (3.9)	6.1 (3.9)	*p* = 0. 975	6.0 (4.1)	6.2 (3.9)	p = 0. 868	6.5 (3.9)	5.9 (3.9)	*p* = 0. 395

*STMI* short-term memory impairement, *ETU* Ebola treatment Unit, *SD* standard-deviation, *F.M.L* Family member loss, *OR* Odds Ratio, *CI* Confidence Interval

^a^Comparison betwen 25–39 and 18–24 group age as far as PTSD was concerned

^b^Comparison betwen ≥40 and 18–24 group age as far as PTSD was concerned

^c^Likelihood chi square test

EVD survivors presenting with STMI were more likely to suffer from depression (OR = 2.440, 95% CI: 1.03, 5.82, *p* = 0.026); and anxiety (OR = 2.24, 95% CI: 1.04, 4.85, *p* = 0.025) compared to STMI free survivors. Survivors suffering from persistent headaches were more likely to exhibit depression (OR = 2.62, 95% CI: 1.12, 6.14, *p* = 0.014) and anxiety (OR = 2.31, 95% CI: 1.06, 5.01, *p* = 0. 020) compared to headache-free survivors (Table 2).

The average time elapsed since discharging from ETU and psychiatric assessment was 6.14 (SD = 3.93) months in PTSD survivors group, 6.03 (SD = 4.07) months in the depression group and 6.52 (SD = 3.97) months in anxiety group. There was no statistically significant relationship between the time elapsed since ETU discharge and the psychiatric disorders we measured among EVD survivors. PTSD, depression and anxiety did not have a statistically significant relationship with educational level or marital status (*p* = 0.102; *p* = 0.489 and *p* = 0. 403) (Table 2).

## Discussion

This study sought to determine the prevalence of PTSD, depression and anxiety among EVD survivors. Moreover it looked to assess the relationship between these psychiatric disorders and other comorbidities among EVD survivors. Symptoms consistent with PTSD, depression and anxiety were found among 24.3%, 24. 3% and 33. 3% of EVD survivors respectively.

It has been shown that understanding psychological reactions among EVD survivors can give opportunity to provide important data about post-treatment Ebola psychological preventative measures [10].

In the cultural context of the studied epidemic, it is quite possible that most EVD survivors did not have sufficient health literacy to easily understand what EVD is, how to avoid it, and how to reach the nearest ETU when their first symptoms appeared, because of their high rate of illiteracy (40.3% had never been to school). This study revealed also that 45.1% EDV survivors had lost loved ones because of EVD, and may have thought they were dying themselves. Many of them did not have an opportunity to mourn with their families or communities. During their hospitalization, they were mostly cared for by foreigners (infectious disease specialists and epidemiologists, who were often expatriate or from other provinces) with whom they did not share the same culture or language. This situation could contribute to psychological distress, during their ETU admission and following discharge. Data collected in Liberia through focus group discussions among EVD survivors revealed that they generally felt stigmatized. The stigma began in the ETU and continued afterwards in the family, community, workplace and even in places of worship [10, 15–17].. Furthermore, some survivors experienced ongoing physical symptoms, which might be a further source of psychological stress. All these stressors are likely to have triggered and maintained PTSD, anxiety and depression symptoms among survivors.

In Sierra Leone, 21% of EVD survivors reported clinically important post traumatic reactions between 3 and 4 weeks post discharge, and these reactions predicted later development of post-traumatic stress disorder [10]. In Guinea, three out of 33 EVD survivors in follow-up program had PTSD symptoms [18]. The psychological impact of EVD is such that it affects even those who have not suffered from it. According to a community based study carried out in Sierra Leone among the general population [19], 16% (95% CI 14.7 to 17.1%) had levels of symptoms consistent with a probable PTSD diagnosis, although the definition and measurement of these symptoms in each study differed.

In this study, factors associated with higher reporting of PTSD symptoms included loss of a family member, persistent headache (those with headache being twice as likely to have PTSD compared to headache-free survivors), and being female. Human gender differences in anxiety and emotional disorder has been reported, with studies generally concluding that women are more likely than men to develop acute stress disorder or PTSD [20, 21]. Some arguments have been made that the increased PTSD prevalence among women is due to a reporting bias because men tend to under-report and women over-report symptoms of PTSD [22], while other authors suggest that this high prevalence is probably due to social expectations related to the male and female gender role; with women expected to be vulnerable, men expected to be tough and more resilient to trauma [23].

Having persistent headaches and losing a loved one could be considered as a cumulative exposure to potentially traumatic experiences, and previous research has elucidated causal links between stress exposure and the development of anxiety disorders [24].

This study showed that the younger the EVD survivor was (18–24 years), the greater was the risk for them to suffer from PTSD. Creamer and Parslow [25] also found that the risk of PTSD was highest between the age of 18 and 24 years for both men and women. Despite what we have found, Norrid et all [26] examined the effects of age on PTSD in a cultural context and compared the effects of of age after similar disaster in three different parts of the world and concluded that PTSD depended upon the social, economic, cultural and historical context of the disaster- stricken-setting, more than it depended on age.

More than one-third of our survivors had persistent headaches and nearly a quarter had short-term memory impairment (STMI). Beni town, where this study was performed, is also an active conflict zone. As discussed before, numerous stressful factors associated with life in the province are probably more likely to trigger anxiety and depression, where there is also an acute mental health services shortage [6].

In this study, symptoms consistent with depression and anxiety were found among 24.3 and 33.3% of EVD survivors respectively. Unsurprisingly, we’ve found that factors associated with higher reporting of anxiety-depression symptoms included STMI and persistent headache (twice more likely to exhibit anxiety-depression symptoms). It is possible that suffering from physical symptoms that some authors consider a « post-Ebola syndrome » - such as STMI and headaches – [27] interferes with daily life and delays social reintegration, in a situation already made tough by stigma [16]. This may then in turn contribute to the development of anxiety and depressive symptoms. Depression is also known to be a common comorbidity of persistent headaches in other populations [28, 29]. As mentioned above, women seem to be more vulnerable to stress- and fear-based disorders, such as anxiety and post-traumatic stress disorder. It has been reported that women are two to three times more likely than men to suffer from generalized anxiety disorders and have higher self-reported anxiety scores [30, 31].

In the same way, anxiety symptoms were mostly reported by female survivors in this study. Since previous research describes existing gender differences in anxiety disorders, in animals models it has been suggested that a conflict anxiety-related serotonergic region that may be particularly vulnerable in females is the midbrain [32]; a role for gonadal hormones, and estrogen in particular, has also been reported [33].

## Limitation of the study

Due to the high illiteracy rate (40.3% EVD survivors had never been to school), EVD survivors needed help in completing the tools used in this study (Posttraumatic *Checklist Scale (PCLS)* and *Hospital Anxiety and Depression Scale (HADS),* even though other study populations have successfully self-completed them in numerous previous studies. Some more difficult-to-understand items were translated by the neuropsychiatrist into Swahili, another commonly used language in the area the study was conducted, to aid understanding. Given the health emergency conditions in which this study was done, it was not possible to conduct a full cross-cultural validation of these scales and the effects of translation upon them.

Furthermore, in the literature, the diagnostic thresholds proposed by various authors were different depending on the populations studied. For this study, we chose a threshold of 44 for PTSD screening and of 8 for the diagnosis of anxiety-depressive disorders as proposed by Bjelland et al. [34]. This PTSD cut-off score is higher than some others proposed, and could have helped to minimize false positive cases [35]. However, to overcome the weakness of this questionnaire, it is often advisable to have more than one structured clinical face-to-face interviews with the patient in order to make an accurate diagnosis. This was not possible in the context of carrying out this work. Patients often had to travel significant distances to meet with health care workers in this program and additional visits were thus not practical.

It also must be mentioned that this cross-sectional study was conducted from October 26 to November 13, 2019 while the EVD outbreak went on up to the April 2020. These results should therefore not be generalized for all EVD survivors.

Finally, this study was conducted in an area where armed conflicts, and their impacts on psychological health, have been ongoing for more than two decades. Some of our patients may have already had PTSD before they suffered from EVD and this was revealed by our screening. Thus our results on psychological trauma may not be fully generalizable to other EVD survivor populations from different contexts.

## Conclusion

Ebola Virus Disease (EVD) is a deadly and feared infectious disease, which can be responsible of debilitating physical and psychological sequelae in survivors including depression and anxiety disorders. Survivors have often seen their loved ones die, and have been confronted with corpses and death. After leaving the hospital, they have to deal with the difficulties of family and socio-professional integration because of stigma associated with the disease. Many also suffer from post-ebola syndromes (headaches, memory disorder, physical pain etc.). These potent psychological and physical stressors may trigger high rates of anxiety disorders and depression, as detected in this study. Surviving EVD is therefore not synonymous with being free from the consequences of the disease, due to this accumulation of stress. This may be even more marked in DRC as the EVD outbreak was declared in areas affected by long lasting armed conflicts.

Implementation of specialized, culturally contextualised programs to sustain psychiatric and psychological care in the Eastern part of the Democratic Republic of Congo should be prioritized by health services battling EVD in the DRC and further research is needed to show how this major burden of physical and psychiatric comorbidity following EVD may be overcome [36, 37].

## Data Availability

All data collected or analysed during the current study are also available from the corresponding author on reasonable request.

## Abbreviations

EVD: Ebola virus disease
ETU: Ebola treatment unit
HADS: Hospital Anxiety and Depression Scale
PCLS: The Posttraumatic Checklist Scale
PTSD: Post-traumatic stress disorder
STMI: Short-term memory impairment
SD: Standard deviation
